# Derivation of Breast Cancer Cell Lines Under Physiological (5%) Oxygen Concentrations

**DOI:** 10.3389/fonc.2018.00425

**Published:** 2018-10-12

**Authors:** Euphemia Y. Leung, Marjan E. Askarian-Amiri, Dean C. Singleton, Carole Ferraro-Peyret, Wayne R. Joseph, Graeme J. Finlay, Reuben J. Broom, Purvi M. Kakadia, Stefan K. Bohlander, Elaine Marshall, Bruce C. Baguley

**Affiliations:** ^1^Auckland Cancer Society Research Centre, University of Auckland, Auckland, New Zealand; ^2^Department of Molecular Medicine and Pathology, University of Auckland, Auckland, New Zealand; ^3^Univ Lyon, Claude Bernard University, Cancer Research Center of Lyon, INSERM 1052, CNRS5286, Faculty of Pharmacy, Lyon, France; ^4^Hospices Civils de Lyon, Molecular Biology of Tumors, GHE Hospital, Bron, France; ^5^Auckland City Hospital—Oncology, Grafton, Auckland, New Zealand

**Keywords:** breast cancer, PI3K mTOR inhibitor BEZ235, estrogen receptor, HER2 inhibitors, 5% and 21% oxygen, CA9, ROS, Triple negative breast cancer (TNBC)

## Abstract

**Background:** Most human breast cancer cell lines currently in use were developed and are cultured under ambient (21%) oxygen conditions. While this is convenient in practical terms, higher ambient oxygen could increase oxygen radical production, potentially modulating signaling pathways. We have derived and grown a series of four human breast cancer cell lines under 5% oxygen, and have compared their properties to those of established breast cancer lines growing under ambient oxygen.

**Methods:** Cell lines were characterized in terms of appearance, cellular DNA content, mutation spectrum, hormone receptor status, pathway utilization and drug sensitivity.

**Results:** Three of the four lines (NZBR1, NZBR2, and NZBR4) were triple negative (ER-, PR-, HER2-), with NZBR1 also over-expressing EGFR. NZBR3 was HER2+ and ER+ and also over-expressed EGFR. Cell lines grown in 5% oxygen showed increased expression of the hypoxia-inducible factor 1 (HIF-1) target gene carbonic anhydrase 9 (*CA9*) and decreased levels of ROS. As determined by protein phosphorylation, NZBR1 showed low AKT pathway utilization while NZBR2 and NZBR4 showed low p70S6K and rpS6 pathway utilization. The lines were characterized for sensitivity to 7-hydroxytamoxifen, doxorubicin, paclitaxel, the PI3K inhibitor BEZ235 and the HER inhibitors lapatinib, afatinib, dacomitinib, and ARRY-380. In some cases they were compared to established breast cancer lines. Of particular note was the high sensitivity of NZBR3 to HER inhibitors. The spectrum of mutations in the NZBR lines was generally similar to that found in commonly used breast cancer cell lines but *TP53* mutations were absent and mutations in *EVI2B, LRP1B*, and *PMS2*, which have not been reported in other breast cancer lines, were detected. The results suggest that the properties of cell lines developed under low oxygen conditions (5% O_2_) are similar to those of commonly used breast cancer cell lines. Although reduced ROS production and increased HIF-1 activity under 5% oxygen can potentially influence experimental outcomes, no difference in sensitivity to estrogen or doxorubicin was observed between cell lines cultured in 5 vs. 21% oxygen.

## Introduction

The humidified atmosphere used in most human tumor cell culture systems includes carbon dioxide and ambient oxygen (21%). This is considerably higher than that *in vivo*, which is thought to average 5% (range 1–8%) as a consequence of oxygen transport, consumption in tissues and diffusion from the blood supply to the tissues (1, 2). The difference raises the question of whether the oxygen concentration in air, for instance by generating increased reactive oxygen species (ROS), might modulate signaling pathways in “regular” cell culture conditions and thus alter responses to therapeutic agents (3, 4). In our previous studies, human melanoma specimens were cultured in an atmosphere of 5% O_2_, 5% CO_2_, 90% N_2_ to minimize oxygen-mediated damage (5) and in subsequent studies, these conditions were used in the development of more than 100 melanoma lines (6, 7) and more than 50 carcinoma lines (8). In this report, we describe four human breast cancer cell lines that were developed under these conditions, to ascertain whether their properties, including receptor status, signaling pathway utilization, mutations and drug responses, were comparable to those of established cell lines.

## Materials and methods

### Cell culture

Culture conditions have been described (9, 10) for MCF-7, SKBr3, MDA-MB-231, BT474, and ZR75.1 cells [purchased from the American Type Culture Collection (ATCC)]. Cell lines were grown in α-modified minimal essential medium (αMEM; Invitrogen) supplemented with 5 μg/mL insulin, 5 μg/mL transferrin and 5 ng/mL sodium selenite (ITS; Roche Applied Sciences), 100 U/mL penicillin, 100 μg/mL streptomycin, and 5% fetal bovine serum (FBS). NZBR1, NZBR2, NZBR3 and NZBR4 cell lines were grown under low oxygen conditions (5% O_2_, 5% CO_2_, 90% N_2_) to mimic physiologically oxygen levels in tumors. The cell lines of ATCC provenance were cultured in an atmosphere of 5% CO_2_ in air at 37°C.

For estrogen response, MCF-7 and NZBR3 cell lines were cultured in estrogen deprived media (phenol red-free RPMI 1640 containing 10% charcoal-stripped fetal bovine serum from ICPbio International Ltd., Auckland, NZ and Invitrogen, Auckland, NZ), and penicillin/streptomycin (10 U/ml and 10 μg/ml, respectively) for the 2 days before 17β-estradiol treatment. Estrogen-deprived medium was used for all assays related to estrogen stimulation.

As described previously (8), tumor tissue taken from four patients undergoing surgery for breast cancer was sent to the histopathologist immediately after surgery, where a portion was placed into growth medium without serum for laboratory studies. Formal consent was obtained from all patients, using guidelines approved by the Northern A Health and Disability Ethics Committee.

Solid tumor specimens were disaggregated either immediately or after overnight storage at 4°C. Normal, adipose, or grossly necrotic material was removed and the tumor tissue was minced finely using crossed scalpels. Tissue was reduced to small clumps by passage through a 0.65-mm stainless steel sieve. The size of the aggregates varied greatly (aggregates of 5–100 cells). Material containing larger aggregates was pipetted into tubes, collected by low-speed centrifugation to remove blood cells, necrotic material, and debris (30 × g, 2 min), and then washed twice (30 × g, 2 min) in growth medium. Preparations were monitored by phase contrast microscopy, and cytospins of cell suspensions were stained by hematoxylin/eosin and examined by a pathologist to ensure that they contained tumor cells. Cultures were set up in growth medium supplemented with 5% FBS under an atmosphere of 5% O_2_, 5% CO_2_, and 90% N_2_ in a Tri-Gas Forma incubator. Tissue culture plates or flasks had previously been coated with a thin layer of agarose to prevent the growth of fibroblasts (5).

Early passage cell lines (< passage 33) used in this study were developed in this laboratory. All cell lines were tested negative for mycoplasma contamination.

### Chemicals and reagents

Everolimus, afatinib, dacomitinib, lapatinib and ARRY-380 was purchased from Selleck Chemicals (Houston, USA). 17β-estradiol, tamoxifen, paclitaxel and doxorubicin were purchased from Sigma (Auckland, NZ). NVP-BEZ235 (11, 12) was synthesized according to published protocols.

### Short tandem repeat profiling

The NZBR cell lines were typed by short tandem repeat profiling by DNA Diagnostics (Auckland, New Zealand) (Table S1). The combination of markers selected was consistent with the National Institute of Standards and Technology database recommendations for identity testing.

### Cell proliferation assay

As described previously (9, 10, 13, 14), proliferation was measured using a thymidine incorporation assay. Cells were seeded (3,000 per well) in 96 well plates in the presence of varying concentrations of inhibitors for 3 days. ^3^H-thymidine (0.04 μCi per well) was added to each well and cultures were incubated for 6 h; cells were harvested on glass fiber filters using an automated TomTec harvester. Filters were incubated with Betaplate Scint and thymidine incorporation measured in a Trilux/Betaplate counter. Effects of inhibitors on the incorporation of ^3^H-thymidine into DNA were determined relative to the control (non-drug-treated) cells.

For the growth response to 17β-estradiol in 5 and 21% oxygen culture conditions, cells were seeded at 2,500 per well in 96 well plates in the presence or absence of 50 nM 17β-estradiol for 3 days. For the growth response to doxorubicin in 5 and 21% oxygen culture conditions, cells were seeded at 1,000 per well in 96 well plates in the presence of varying concentrations of inhibitors for 5 days.

### Measurement of reactive oxygen species (ROS)

Intracellular ROS were detected with the cell-permeable fluorescent probe 2',7'- dichlorofluorescein diacetate (DCFDA) (ABCAM) according to the manufacturer's instruction. Cells (1 × 10^5^) were harvested, incubated with 20 μM DCFDA in medium for 60 min in the dark at 37°C. Cells were analyzed in a BD Accuri™ Flow Cytometer.

### Reverse transcription, cDNA synthesis, and quantitative PCR

As described in detail (15), oligo-dT and random primers were used to reverse transcribe RNA with qScript Flex cDNA kit (Quantabio) according to the manufacturer's instructions. To investigate whether estrogen exposure can lead to increased expression of estrogen response genes and whether cell lines grown in 5% oxygen conditions can induce carbonic anhydrase IX (*CA9*), which is the most well established target of HIF, quantitative RT-PCR (qRT-PCR) was performed using gene-specific primers (Table S2) and Sybr Green Master Mix (Life Technologies), and expression values were normalized relative to *HPRT1* RNA expression. The 2^∧^(–delta delta CT) method was used to analyze the relative changes in gene expression.

### Western blotting

As described (9, 10, 16), breast cancer cell lines were grown to log-phase, washed twice with ice-cold PBS, and lysed in SDS lysis buffer according to the manufacturer's protocol (Cell Signaling Technology, Danvers, MA). Protein concentration was quantified using the bicinchoninic acid reagent (Sigma). Cell lysates containing 25 μg of protein were separated by SDS-polyacrylamide gel electrophoresis (PAGE), and transferred to PVDF membranes (Millipore). Membranes were immunoblotted with antibodies against phospho-AKT (S473), total AKT, phospho-p70S6K (T389), total p70S6K, phospho-rpS6 (S235/ 236), total rpS6, phospho-ERK (T202/Y204), total ERK (all from Cell Signaling Technology), tubulin (Sigma), and actin (Millipore). Bound antibody was visualized using SuperSignal West Pico (Thermo Scientific, Waltham, MA) or ECL plus (GE Healthcare, Auckland, NZ) and the chemiluminescence detection system by Fujifilm Las-3000.

### Genomic analysis

For whole exome sequencing (WES), 250 ng of genomic DNA from each cell line was sheared using the EpiShear™ Multi-Sample Sonicator (Active Motif). The quantity and fragment size of the sheared DNA were assessed on a Tapestation 2200 (Agilent) with the high sensitivity D1000 tape. Sheared DNA (100 ng) was used for the preparation of the whole exome libraries (WELs). WELs were prepared using the SureSelect XT2 (SSXT2) reagent kit and the SureSelect Clinical Research Exome V2 exome enrichment kit following the manufacturer's instructions (Agilent Technologies). The WELs were sequenced on a NextSeq500 (NCS v2.0, Illumina Inc.) to obtain around 40 to 44 million paired end reads (2 × 150 bp, 12 to 13 Gbp) per exome.

The quality of the sequences was assessed using Fastqc (https://www.bioinformatics.babraham.ac.uk/projects/fastqc/). The reads were aligned to the human reference genome (hg19) with BWA (bwa 0.7.12) (17). The resulting sam files were converted to bam and then the bam files were sorted using the samtools (Samtools-1.3.1) (18). Mpileup files were generated (Samtools-1.3.1) with the following parameters: maximum depth (-d) 500, minimum base quality (-Q) 15 and minimum mapping quality (-q) 10. Varscan v2.3.9 (19) was used to call variants and to generate VCF (variant call format) files (20). The variants in the vcf files were annotated with information from various SNP databases (dbSNP138 etc) using ANNOVAR (21) followed by the annotation for the variants' effect with SnpEffect (22). Variants present in the 1000genome_Oct2014 database were excluded. Variants predicted to have a “High” (e.g., nonsense) or “Moderate” (missense) impact in genes in the two breast cancer gene lists (see Results Section) were selected using SnpSift (23).

### Data analysis

*T*-tests or Mann-Whitney Rank Sum Tests was used for comparison of groups. Correlation analysis was performed with Spearman Rank Order correlation coefficient (R) and statistical significance (P) using SigmaPlot (Systat Software, Inc.). Values of *P* < 0.05 were considered to be statistically significant.

## Results

### Initial characterization of NZ breast cancer cell lines

**Figure 1:**
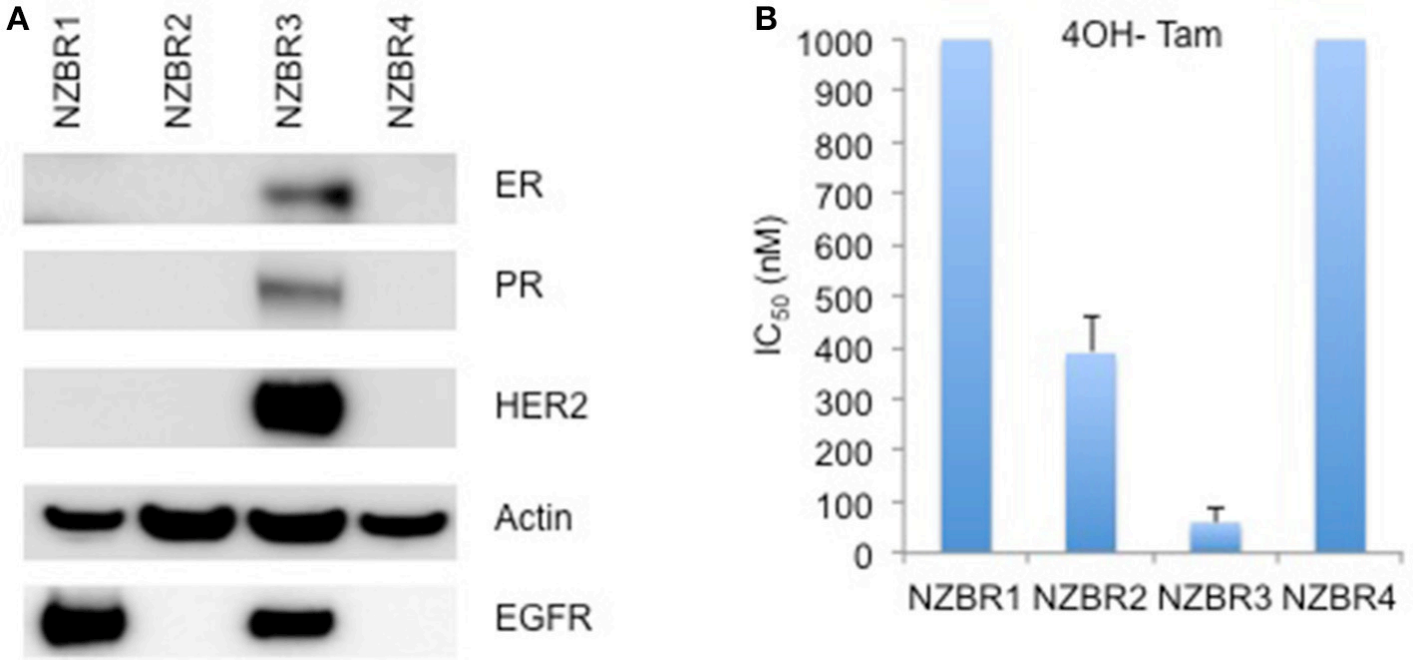
Receptor expression, and growth inhibitory concentrations for NZBR cell lines exposed to tamoxifen. **(A)** Relative expression of estrogen receptor (ER), progesterone receptor (PR), HER2 and EGFR in NZBR1, NZBR2, NZBR3, and NZBR4 breast cancer cell lines. Actin is shown as loading control. **(B)** IC50 values (50% inhibition of growth) for tamoxifen, shown as the mean ± standard error of triplicate experiments. The highest tamoxifen concentration used (1,000 nM) is indicated where 50% growth inhibition was not reached.

The cell lines were characterized by cellular DNA content, hormone receptor expression and tamoxifen sensitivity. The lines were all aneuploid and three of the four lines (NZBR1, NZBR2, and NZBR4) were triple-negative with no expression of estrogen receptor, progesterone receptor and HER2 (Table 1; Figure 1A). The ER+ NZBR3 cell line was sensitive to tamoxifen with an IC50 value of 60 nM (Figure 1B). The triple-negative cell lines were relatively resistant to tamoxifen with IC50 values of >1000, 390, and >1000 nM, respectively.

**Table 1:** Source, clinical and pathological features of tumors used to derive the New Zealand Breast Cancer cell lines, and DNA ploidy.

**Cell line**	**Tumor type**	**Age**	**Ploidy**
NZBR1	IC	58	1.63
NZBR2	IDC	40	1.12
NZBR3	IDC	72	1.42
NZBR4	ND	ND	1.5

*IC, infiltrating carcinoma; IDC, infiltrating ductal carcinoma; Ploidy, DNA content relative to diploid; ND, no data*.

### Sensitivity of ER+ MCF-7 and NZBR3 to estrogen

**Figure 2:**
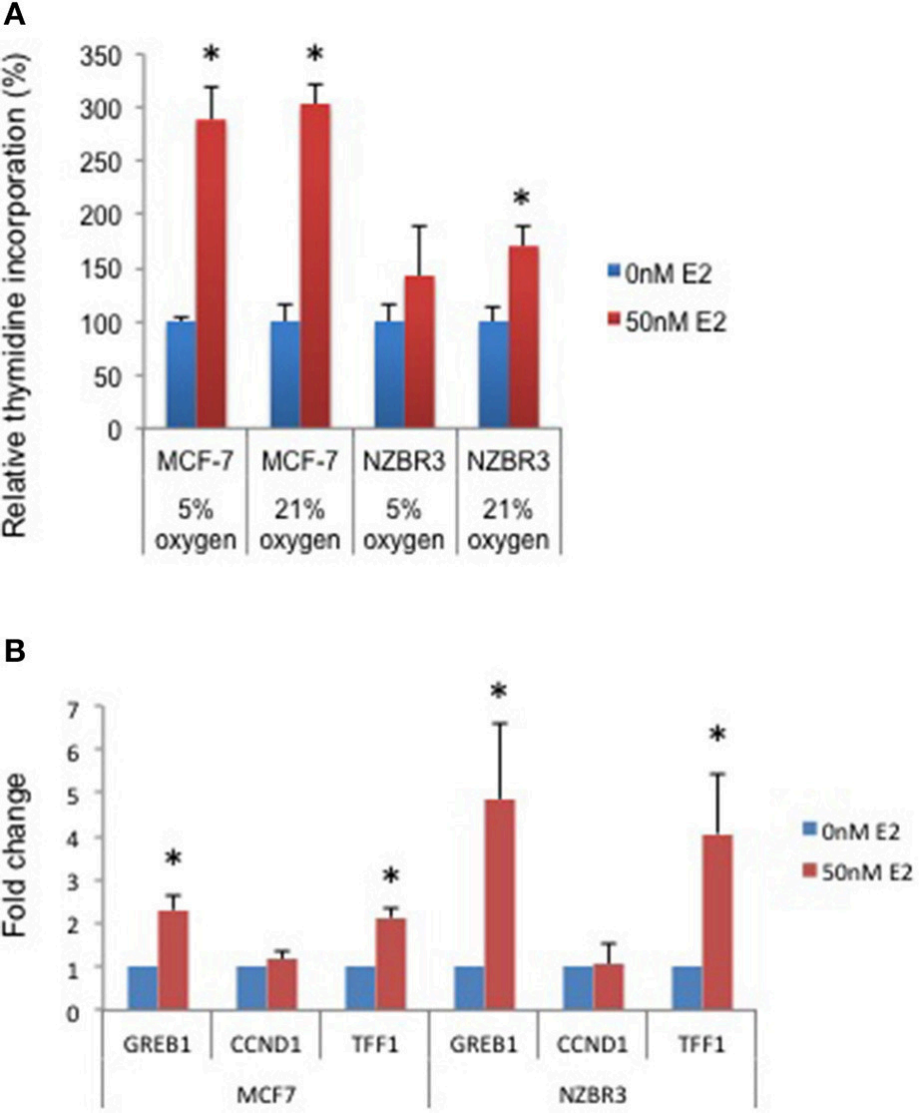
Sensitivity of ER + MCF-7 and NZBR3 cells to estrogen. **(A)** Relative growth of MCF-7 and NZBR3 cells in response to 17β-estradiol was measured using thymidine incorporation when cells were cultured under 5 or 21% oxygen conditions. **(B)** Comparison of estrogen response gene (*GREB1, CCND1*, and *TFF1*) expression measured by quantitative RT-PCR in breast cancer cells exposed to 17β-estradiol (50 nM) for 24 h. Results are shown as the mean ± standard error of triplicate experiments. ^*^p < 0.05.

A [^3^H]-thymidine incorporation assay was used to assess the effect of estrogen on cell proliferation when the cells were cultured under 5 and 21% oxygen conditions. Both MCF-7 and NZBR3 cell lines displayed significant growth stimulation by estrogen and MCF-7 cells showed increased response to estrogen as compared to NZBR3. However, the two oxygen concentrations did not have distinguishable effects on the growth response to estrogen (Figure 2A).

We next examined the expression of three estrogen-responsive genes in these two cell lines; namely Growth Regulation By Estrogen In Breast Cancer 1 (*GREB1*), Cyclin D1 (*CCND1*) and Trefoil Factor 1 (*TTF1*) (24). Both *GREB1* and *TFF1* showed significant upregulation with estrogen stimulation (Figure 2B).

### Comparison of reactive oxygen species (ROS) production in cells cultured under 5 and 21% oxygen condition

**Figure 3:**
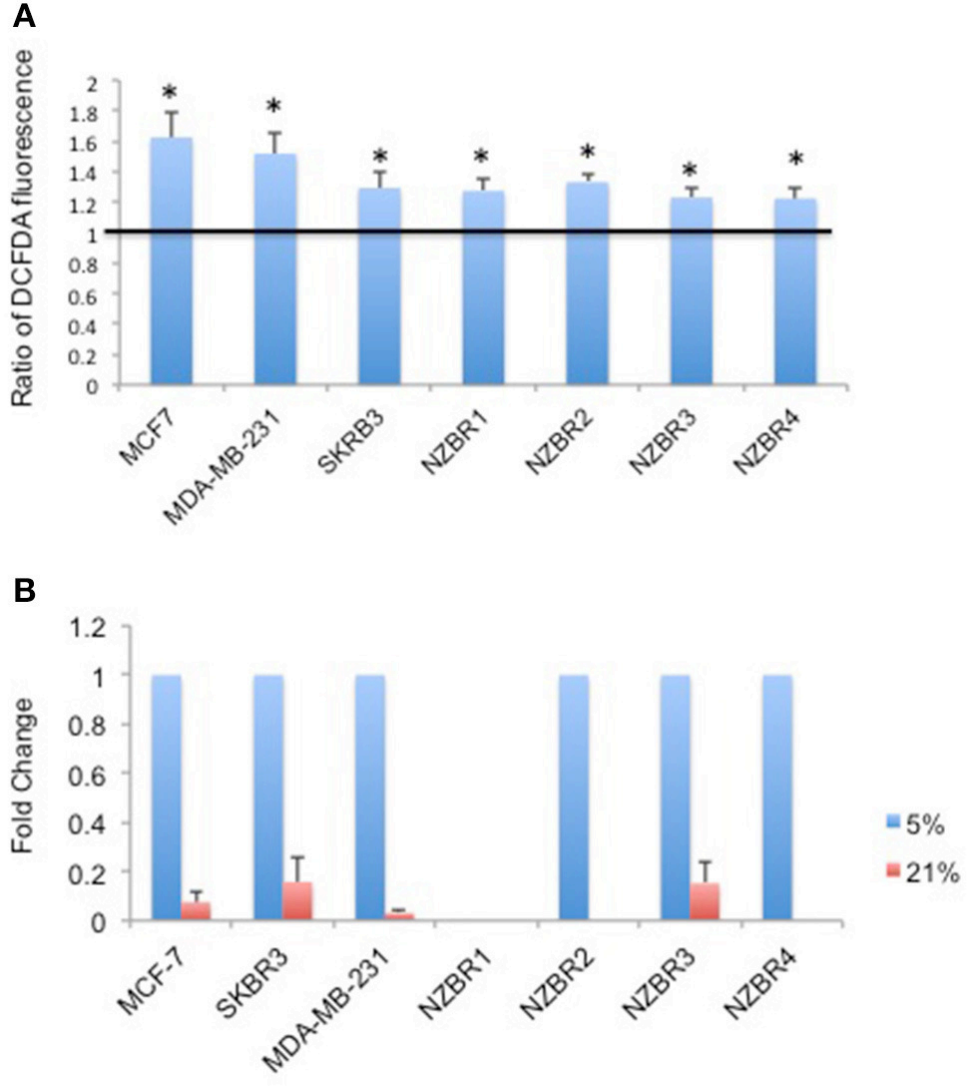
Differential response to culture conditions of 5 and 21% oxygen. **(A)** Relative ROS concentration in cells cultured under 5 or 21% oxygen conditions as measured by DCFDA fluorescence. **(B)** Comparison of hypoxia target gene *CA9* expression measured by quantitative RT-PCR in breast cancer cells under 5 or 21% oxygen conditions. Results are shown as the mean ± standard error of triplicate experiments. ^*^p < 0.05.

As ROS production can be affected by the oxygen concentration during culture, we measured intracellular ROS levels by using the hydrogen peroxide-sensitive dye 2′,7′ –dichlorofluorescein diacetate (DCFDA) in cells cultured under 5 and 21% oxygen concentrations. A significant increase in ROS concentrations was observed in cells cultured in 21% oxygen (pairwise *T*- test, *p* < 0.05) (Figure 3A).

### Expression of hypoxia inducible factor 1-regulated carbonic anhydrase IX (*CA9*) under 5 and 21% oxygen conditions

Oxygen concentration can affect the expression of hypoxia-inducible factor 1 (HIF-1)-regulated genes, including carbonic anhydrase IX (*CA9*) (25). Cell lines were cultured at oxygen concentrations of 5 and 21% for over 2 weeks before testing for the expression of *CA9*. Apart from NZBR1, which had no measurable transcript by RT-qPCR, all cell lines showed decreased *CA9* expression in 21% compared with 5% oxygen (Figure 3B).

### mTOR pathway signaling and sensitivity to mTOR inhibitor everolimus and dual PI3K and mTOR inhibitor BEZ235

**Figure 4:**
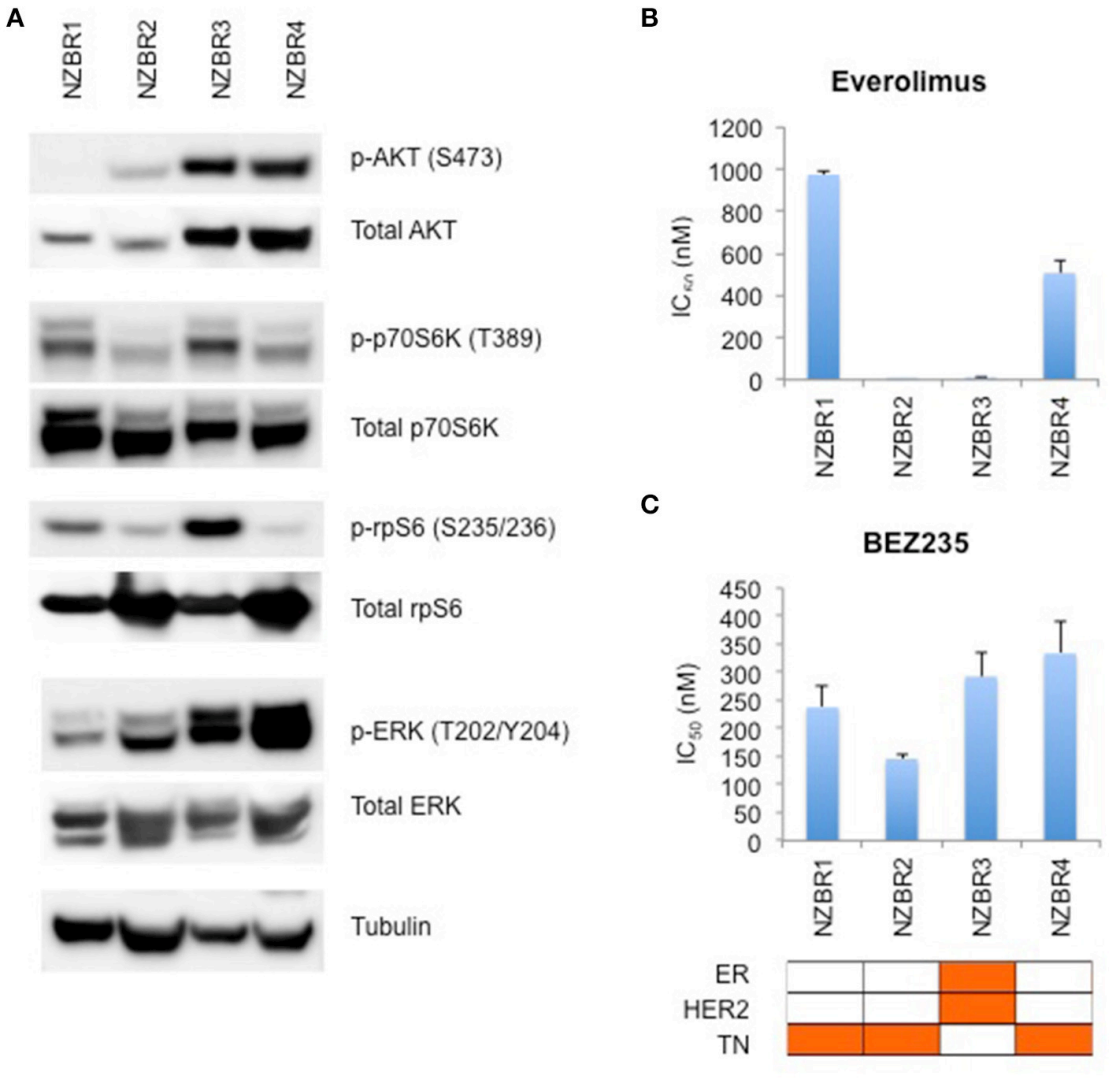
Signaling pathway usage and growth inhibitory concentrations for NZBR cell lines exposed to different drugs. **(A)** Signaling pathway usage as measured by phosphorylation of AKT, p70S6K, rpS6 and ERK. Immunoblots with antibodies specific for phosphorylated proteins and their respective total protein are indicated. Tubulin is the loading control. IC50 values for **(B)** everolimus, and **(C)** NVP-BEZ235, shown as the mean ± standard error of triplicate experiments. Cell lines are identified as estrogen receptor (ER) positive, HER2 positive and triple negative (TN) (orange, here and below).

The phosphorylation status of AKT, p70S6K, rpS6, and ERK in the NZBR lines was examined (Figure 4A). NZBR1 showed the lowest degree of phosphorylated AKT expression, yet both NZBR1 and NBZR3 had comparable levels of phosphorylated p70S6K (mTOR pathway effector). On the other hand, both NZBR2 and NZBR4 contained low levels of phosphorylated p70S6K, indicating that they had low mTOR pathway utilization. NZBR2 and NZBR4 showed a low degree of phosphorylated rpS6, whereas NZBR1 had low levels of phosphorylated ERK.

We have shown that estrogen receptor positive (ER+) breast cancer cell lines generally are more sensitive to everolimus than are receptor negative lines (9). However, ER expression is not a requirement for an everolimus response since some ER negative breast cancer cell lines are sensitive to growth inhibition by everolimus (9), as exemplified here by the TN NZBR2 cells, which are exquisitely sensitive to everolimus (Figure 4B). The degree of signaling via the PI3K/AKT/mTOR pathway did not correlate with growth inhibitory responses to everolimus or BEZ235 treatment (Figures 4B,C).

Two out of the three triple-negative NZBR cell lines were resistant to everolimus, with IC50 values of over 500 nM (Figure 4B) in a 3-day ^3^H-thymidine incorporation assay. The ER+ HER2+ NZBR3 cells showed high sensitivity to everolimus (IC50 11.5 nM), yet the triple-negative NZBR2 with low mTOR pathway utilization also showed increased everolimus sensitivity (IC50 1.1 nM) (Figure 4B). Significant differences (*p* < 0.05) between NZBR2 and the other three cell lines in BEZ235 sensitivity were also observed.

### Sensitivity to paclitaxel and doxorubicin

**Figure 5:**
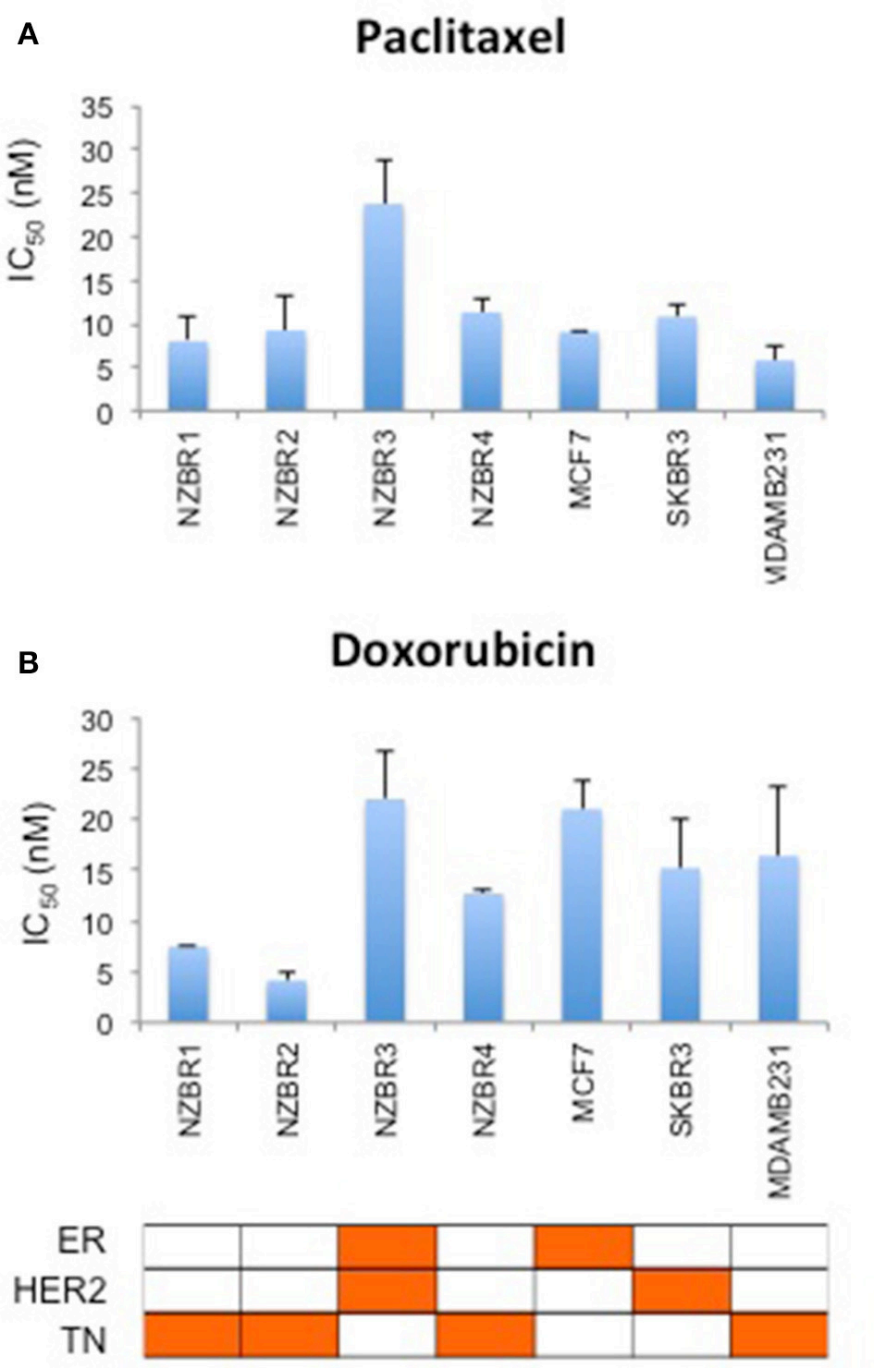
Sensitivity of NZBR cell lines to cytotoxic drugs. IC50 values for **(A)** paclitaxel and **(B)** doxorubicin, shown as the mean ± standard error of triplicate experiments.

The sensitivities of the NZBR cell lines, and the ER+ MCF-7, HER2+ SKBR3 and triple-negative MDA-MB-231 cell lines to the therapeutic agents doxorubicin (a topoisomerase II poison) and paclitaxel (a microtubule poison) were tested (Figure 5). The ER+ HER2+ NZBR3 cell line was least sensitive of all the cell lines to both paclitaxel and doxorubicin.

Since doxorubicin-induced release of free radicals may cause oxidative stress (26), we compared the sensitivity to doxorubicin when the cell lines were cultured under 5 vs. 21% oxygen conditions. No significant difference (pairwise *T*-test, *p* > 0.05) in sensitivity to doxorubicin was observed when the cell lines were cultured in 5% vs. 21% oxygen (Figure S1).

### Sensitivity to HER2 inhibitors lapatinib, afatinib, dacomitinib and ARRY-380

**Figure 6:**
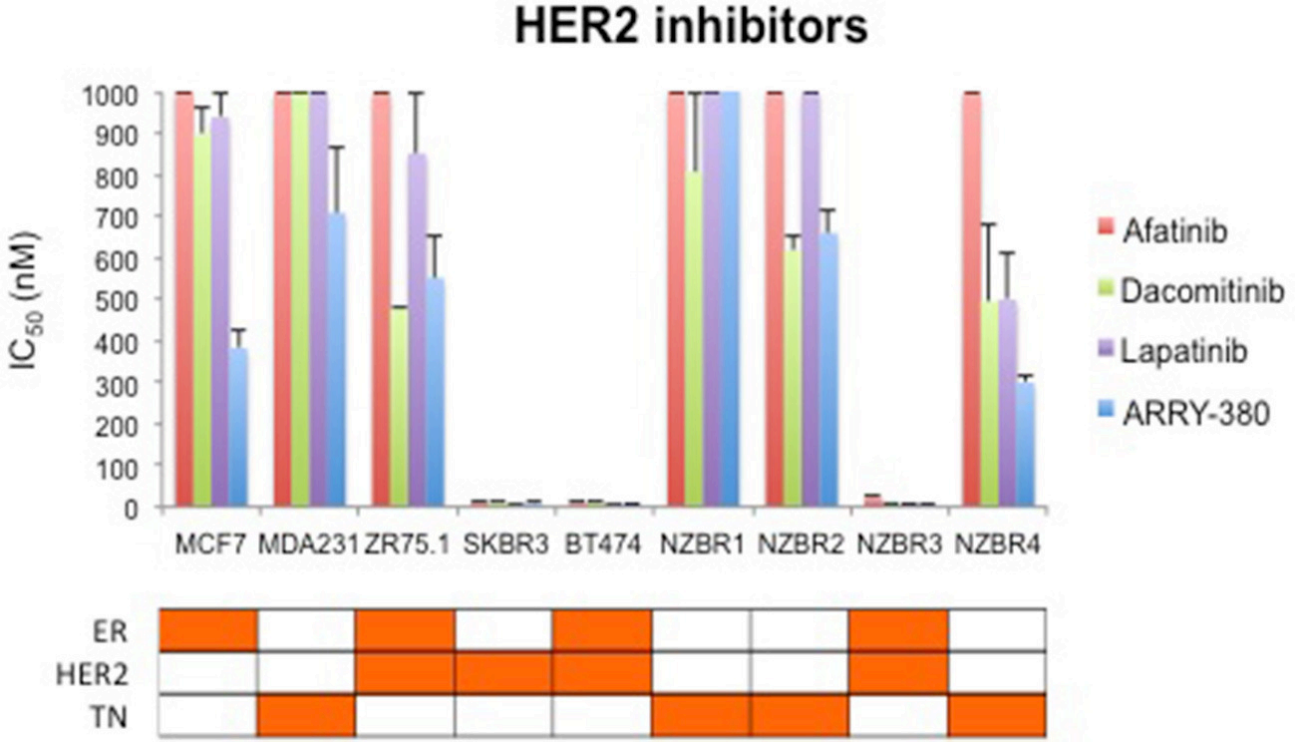
Sensitivity of breast cancer cell lines exposed to kinase inhibitors. IC50 values for afatinib, dacomitinib, lapatinib and ARRY-380, shown as the mean ± standard error of triplicate experiments. The highest inhibitor concentration used (1,000 nM) is indicated where 50% growth inhibition was not reached.

We compared the sensitivity of the NZBR cell lines and other breast cancer cell lines with or without ER or HER2 to the HER2 inhibitors lapatinib, afatinib, dacomitinib and ARRY-380 (ONT-380; irbinitinib; tuncatinib). As expected, drug resistance was observed in the HER2- cell lines; however, the HER2+ cell line ZR75.1 also showed similar resistance (Figure 6). HER2+ NZBR3 cells showed high sensitivity to all HER2 inhibitors tested. These data indicate that HER2 expression is necessary but not sufficient for augmented sensitivity to HER2 inhibitors. HER2 inhibitor sensitivity was not dependent on co-expression of ER.

### Effects of ARRY-380 on signal transduction in four HER2+ breast cancer cell lines

**Figure 7:**
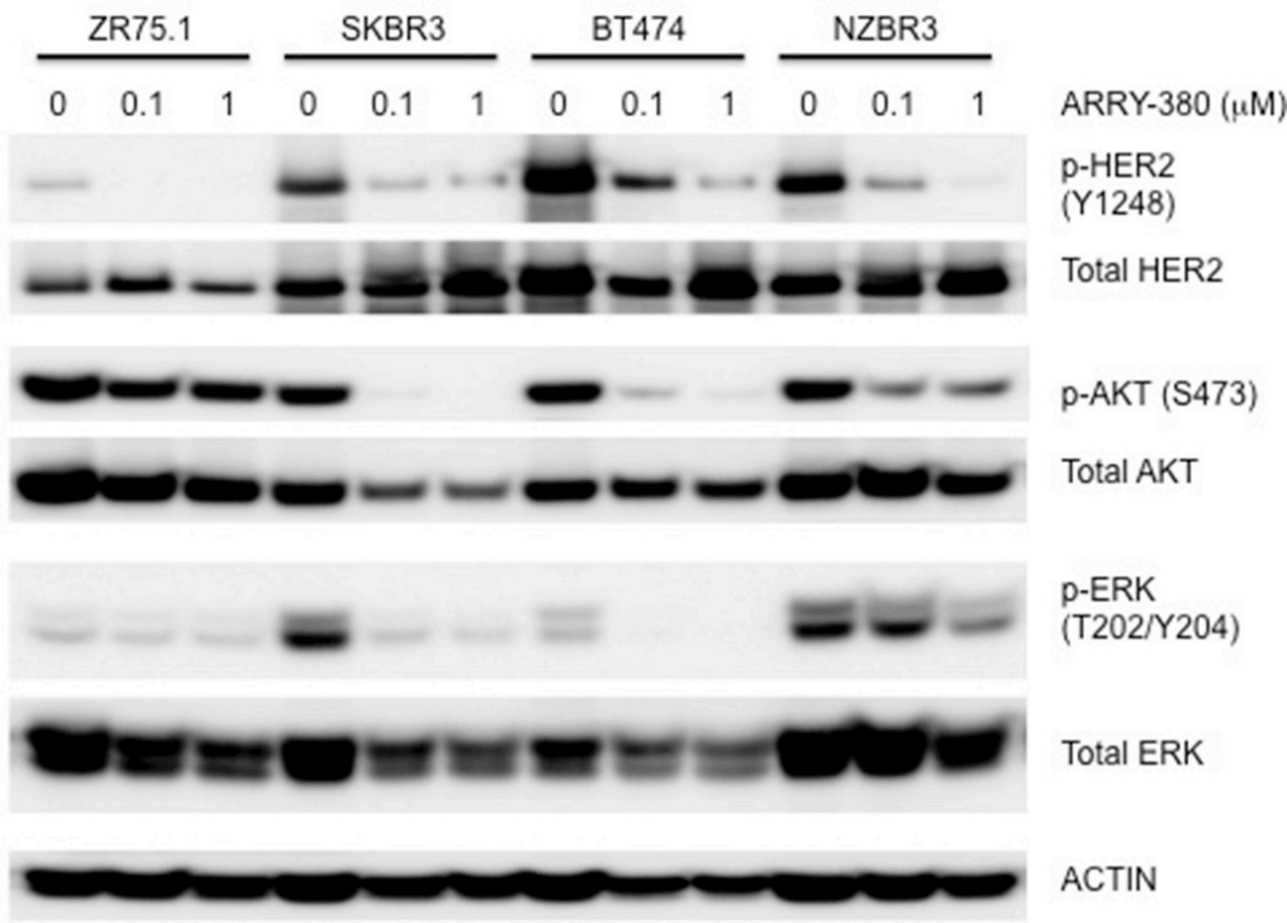
Sensitivity of signaling pathways in ZR75.1, SKBR3, BT474 and NZBR3 breast cancer cell lines to ARRY-380. Cells were treated with ARRY-380 for 24 h and signaling pathway usage was measured by phosphorylation of HER2, AKT, and ERK. Immunoblots with antibodies specific for phosphorylated proteins are indicated. Actin is the loading control.

The effects of ARRY-380 on HER2, AKT, and ERK activation were examined in four HER2+ breast cancer cell lines, which showed a range of sensitivities to ARRY-380. After overnight exposure to 100 nM or 1000 nM of ARRY-380, we observed no effect on total HER2, AKT and ERK expression in the cell lines. ARRY-380 inhibited HER2 phosphorylation in all lines, and ERK phosphorylation in cell lines with detectable phosphorylation (Figure 7). Of interest, the HER2 inhibitor-resistant ZR75.1 cell line showed minimal suppression of AKT phosphorylation by ARRY-380 as compared to the other three HER2 inhibitor sensitive lines (Figures 6, 7). Even though ZR75.1 cells showed loss of HER2 phosphorylation in response to ARRY-380, this was not accompanied by any diminution of the phosphorylation of ERK.

### Genomic analysis

To examine the mutation status of these cell lines, the following genes (*AKT1, ATM, BARD1, BRCA1, BRCA2, BRIP1, CDH1, CHEK2, EPCAM, FAM175A, FANCC, MEN1, MRE11A, MLH1, MSH2, MSH6, MUTYH, NBN, NF1, ALB2, PIK3CA, PMS1, PMS2, PTEN, RAD50, RAD51C, RAD51D, STK11, TP53*, and *XRCCC2*) were selected from the hereditary breast cancer panel of AmbryGenetics, GeneDx, and University of Washington. The somatic mutations gene list (*GATA3, ESR1, TBX3, RUNX1, NCOR1, KMT2C, SPEN, ARID1A, NOTCH2, MAP2K4, RB1, MTOR, MED12, and LRP1B*) was selected from somatic mutations in the cancer (COSMIC) database. Several sequence variants that result in missense or nonsense mutations were detected in genes associated with breast cancer (Table 2). Since germline DNA samples for these cell lines were not available, it is not known whether these mutations were of somatic or germline origin. We detected mutations in *BRCA2, AKT1, NF1, EVI2B, MRE11A, ATM, BRCA2, MLH1, PMS2*, and *LRP1B* (Table 2).

**Table 2:** Nine genes with mutations in the New Zealand breast cancer cell lines.

**Cell line**	**Gene**	**Mutation**		**Impact**	**Number of times reported**
		**Nucleotide**	**Amino acids**		
NZBR2	BRCA2	c.6070C > A,	Q2024K	High	25
	AKT1	c.1234G > A	V412M	High	12
NZBR3	NF1	c.932G > A	G311E	Moderate	15
	EVI2B	c.881C > G	S294^*^	High	ND
	LRP1B	c.10567G > C	A3523P	Moderate	4
		c.3578G > C	R1193T	Moderate	
NZBR4	MRE11A	c.1394T > G	V465G	Moderate	1
	PMS2	c.977C > T	S326F	Moderate	1
	MLH1	c.1460G >C	R487P	Moderate	1
		c.1852A > G	K618E	Moderate	
		c.1853A > C	K618T	Moderate	
		c.977C > T	S326F	Moderate	
	ATM	c.2672C > G	S891^*^	High	21
	BRCA2	c.9976A > T	K3326^*^	High	25

*Among the mutations found in NZBR lines, only the BRCA2 c.9976A > T mutation has previously been reported in the breast carcinoma dataset from cBioPortal using The Cancer Genome Atlas (TCGA) Breast Invasive Carcinoma project (n = 825 cases) from Nature 2012 (27)*.

* *, stop codon*.

Most mutated genes found in the NZBR cell lines had also been found to be mutated in other breast cancer cell lines (Table 3). The mutation frequencies detected in comparison to those of previously characterized breast cancer cell lines and breast carcinomas are listed in Table S3. The spectrum of mutations was generally similar to that found in commonly used breast cancer cell lines, although *EVI2B, LRP1B*, and *PMS2* mutations were not found in other breast cancer cell lines. (Table 3). Since the *EVI2B* (Ectopic viral integration site 2B protein homolog) gene lies within an intron of the *NF1* (Neurofibromatosis type 1) gene and is transcribed in the opposite direction to the *NF1* gene (28), we compared *NF1* and *EVI2B* RNA expression levels in relation to those of the estrogen receptor gene *ESR1* in the genome-wide RNA transcript profile from TCGA (breast invasive carcinoma gene expression) using the RNAseq data set (TCGA_BRCA_exp_HiSeqV2-2017-09-08; in 1,218 tumor tissue samples). A weak but significant positive correlation was observed in the expression of *ESR1* and *NF1* (Spearman's order coefficient *r* = 0.313, *p* = 0.0000002) while *EVI2B* and *ESR1* expression were negatively correlated (*r* = −0.178, *p* = 4.4 × 10^−10^). No significant correlation of *NF1* and *EVI2B* was observed (*r* = −0.002, *p* = 0.927).

**Table 3:** Mutated genes of New Zealand breast cancer cell lines found in other breast cancer cell lines.

**Mutation**	**Cell line**	**Protein change**	**Mutation type**
*AKT1*	MDA-MB-361	D3N	Missense
*ATM*	HCC1500	W2638^*^	Nonsense
	BT474	E2468K	Missense
	EVSAT	L1472F	Missense
	HCC1569	L1824F	Missense
	MDA-MB-453	E1856Q	Missense
*BRCA2*	HCC1395	E1593^*^	Nonsense
	HCC1569	V1862^*^	Frame Shift Deletion
	HCC1599	K1517Ifs^*^23	Frame Shift Deletion
	BT474	S3094^*^	Nonsense Mutation
	BT20	T3357R	Missense
	HCC1569	N1100T	Missense
	MDA-MB-361	N1657S	Missense
	ZR7530	M2322I	Missense
*EVI2B*	ND		
*LRP1B*	ND		
*MLH1*	BT20	E578G	Missense
	HCC1428	D132H	Missense
*MRE11A*	HCC2218	H302Y	Missense
	MDA-MB-134VI	R380C	Missense
*NF1*	BT483	R1204W	Missense
	HCC202	X465_splice	Splice Site
	MDA-MB-231	T467Hfs^*^3	Frame Shift Insertion
	EFM192A	Q347H	Missense
	MDA-MB-468	Q1033R	Missense
	UACC893	Q1033R	Missense
	UACC893	F1037C	Missense
	HS578T	G2745R	Missense
*PMS2*	ND		

Cell lines with mutated genes from cBioPortal (www.cbioportal.org) using Cancer Cell Line Encyclopedia (Novartis/Broad, Nature 2012) breast cancer cell lines (n = 56). ND, no data;

* *, stop codon*.

## Discussion

Our findings show that the behavior of the four breast cancer cell lines developed under low oxygen conditions (5% O_2_) is generally similar to that of commercially available breast cancer cell lines. It is of interest that the hormone receptor status of the new lines covers the main classes of breast cancer, with three of the four having triple negative receptor status. Proliferation of the ER+ NZBR3 cell line was sensitive to estrogen stimulation, and expression of the estrogen responsive genes *GREB1* and *TFF1* was increased after 24 h of estrogen exposure (Figure 2). As the cyclin D1 response decreased to background levels by 24 h, the lack of upregulation of *CCDN1* in our study is consistent with reports by others (29).

Apart from NZBR1, the remaining three NZBR cell lines, MCF-7, SKBR3, and MDA-MB-231 showed higher expression of hypoxia-inducible factor-regulated gene *CA9* (30) when cultured under 5% oxygen conditions. Significantly increased ROS levels were also found in cell lines cultured under 21% oxygen conditions (Figure 3A). It has been suggested that abundant ROS and associated oxidative stress in cells cultured at high oxygen levels could affect the physiology of cells in culture (3, 31), which could potentially influence cell phenotypes and experimental outcomes.

The genomic mutation frequency in the breast cancer cell lines is significantly higher than that in breast carcinoma *in vivo* (*T* test; *p* < 0.002) (Table S3), but the study size is too small to make conclusions about the relative mutation frequencies of the low oxygen-derived lines and ambient oxygen-derived lines. The spectrum of mutations in the NZBR lines is similar to that found in commonly used breast cancer cell lines, except that the *EVI2B, LRP1B* and *PMS2* mutations have not been reported in breast cancer lines (Table 3). The availability of sequence data for these cell lines allows investigation of relationships to drug sensitivity. A surprising finding was that no *TP53* mutations were identified. *TP53* mutations were identified in 18/32 lines (56%) of a cell line cohort (32) and in 23% of the breast cancer samples (33). However, the number of cell lines generated in this study is too small to conclude that the frequency of *TP53* mutations is lower in cell lines derived under low oxygen conditions.

The triple negative line NZBR1 is of interest because of the relative absence of gene mutations in the genome sequence. One possible explanation is that this cell line possesses a CpG island methylator phenotype (CIMP) (14–16) that contributes to its neoplastic properties (34, 35). NZBR1 expresses EGFR, but it may not depend on the EGFR pathway for survival since it is insensitive to all the HER2 inhibitors tested and these inhibitors also inhibit the EGFR. NZBR1 is generally resistant to targeted agents but is moderately sensitive to the cytotoxic drugs doxorubicin and paclitaxel.

The triple negative line NZBR2 carries a *BRCA2* mutation as well as an *AKT1* mutation. The latter differs from the hot spot mutation AKT1^E12K^ that confers sensitivity to the dual mTOR/PI3K inhibitor BEZ235 (36); and NZBR2 shows only moderate sensitivity to BEZ235. NZBR2 shows high sensitivity to everolimus, which is approved for treating advanced hormone receptor-positive, HER2-negative breast cancer. NZBR2 is sensitive to everolimus despite its low level of p70S6K phosphorylation. This result extends our previous study indicating that everolimus sensitivity does not require ER signaling (9).

The ER+ NZBR3 cell line expresses HER2 and is highly sensitive to tamoxifen, but is relatively resistant to the cytotoxic drugs paclitaxel and doxorubicin. It shows high phospho-AKT expression, suggesting sensitivity to AKT inhibitors (37). NZBR3 is also highly responsive to the HER2 inhibitors lapatinib, afatinib, dacomitinib, and ARRY-380. Mutations for *EVI2B, LRP1B*, and *NF1* have also been identified (Table 2). EVI2B is a transmembrane protein (28) while *NF1* is a tumor suppressor gene (38). *NF1* loss is associated with resistance to BRAF inhibitors (39) and EGFR inhibitors (40). The *EVI2B* gene lies within an intron of the *NF1* gene and is transcribed in the opposite direction to the *NF1* gene, but their expression is not correlated, suggesting that they are independently regulated. *NF1* mutations are associated with increased ERK phosphorylation (41), RAF-MEK-ERK signaling and phosphoinositide 3-kinase (PI3K)/mTOR pathway utilization (42). Patients whose cancers, including breast cancers, possess *NF1* mutations have worse survival (43, 44). This may be consistent with the relative resistance of NZBR3 cells to paclitaxel and doxorubicin (Figure 5). However, these cells are sensitive to HER2 inhibitors, suggesting that they depend more on AKT signaling (which is sensitive to HER2 inhibitors) than on RAS/NF1-ERK signaling (which is less sensitive; Figure 7). *LRP1B* is a putative tumor suppressor and a member of the low-density lipoprotein receptor family (45); gene mutation has been reported in several cancer types but not previously in breast cancer (46–48).

The triple negative NZBR4 line is interesting in that it has a number of gene mutations associated with DNA repair including *BRCA2*, the double strand break repair nuclease homolog A (*MRE11A*), mismatch repair system component homolog 2 (*PMS2*), MutL homolog 1 (*MLH1*) and Ataxia Telangiectasia Mutated serine/threonine kinase (*ATM*). *BRCA2*, a tumor suppressor that is essential for the repair of double-strand DNA breaks by homologous recombination, is associated with risk of developing breast cancer (49–52). MRE11A is part of the DNA double-strand break repair MRE11/RAD50/NBS1 complex that is required for non-homologous joining of DNA ends which associates with breast cancer risk (53). Heterozygous germline mutations in the mismatch repair genes including *MLH1* and *PMS2* can cause Lynch syndrome, an autosomal dominant cancer predisposition syndrome conferring a high risk of colorectal, endometrial, and other cancers in adulthood (54, 55). Mutations of *MLH1* and *PMS2* contribute to microsatellite instability and increased mutation rates in cancer cells (56). The *ATM* gene encodes a protein kinase that activates the response to double strand DNA breaks. Pathological variants of this gene showed a significantly increased risk of breast cancer with a penetrance that appears similar to that conferred by germline mutations in *BRCA2* (57). It is possible that mutations of genes associated with the DNA repair pathways may render NZBR4 cells responsive to PARP inhibitors (58).

The drug sensitivity profiles reported here include comparative data for a series of HER2 inhibitors. Lapatinib is an EGFR and HER2 inhibitor with IC50s of 10.8 and 9.2 nM in cell-free assays. Afatinib is an inhibitor of EGFR (wt), EGFR (L858R), EGFR (L858R/T790M) and HER2 with IC50s of 0.5, 0.4, 10, and 14 nM respectively in cell-free assays. Dacomitinib is a potent, irreversible pan-ErbB inhibitor, mostly of EGFR with an IC50 value of 6 nM in a cell-free assay. ARRY-380 is a potent and selective HER2 inhibitor with IC50 of 8 nM, equipotent against truncated p95-HER2, 500-fold more selective for HER2 over EGFR (Selleckchem.com; all enzyme inhibition data). ARRY-380 is under clinical investigation for treating HER2+ metastatic breast cancer patients. Since ARRY-380 is HER2 specific, it has the potential to block HER2 signaling without causing the toxicities of EGFR inhibition (59). In comparing the responses of the NZBR cell lines to HER2 inhibitors, we have included data for four established lines, MCF-7, SKBR3, MDA-MB-231, and ZR75.1. ARRY-380, the most effective of the HER inhibitors tested, is not active against the HER2+ cell line ZR75.1, which is also resistant to other HER2-directed inhibitors (Figure 6). However, ZR75.1 cells have high levels of phosphorylated AKT and low levels of phosphorylated HER2, and ARRY-380 exposure (up to 1 μM) failed to suppress the relative abundance of phosphorylated AKT. Since the HER2 pathway is weakly active, the results suggest that an alternative signaling pathway can contribute to growth and survival. ZR75.1 cells are also highly sensitive to the mTOR inhibitor everolimus (9) and it is possible that combination of everolimus with a HER2 inhibitor could reduce the rate of acquired drug resistance. Understanding the mechanisms that contribute to HER2 inhibitor resistance, including identification of predictive biomarkers such as HER2 phosphorylation status, is important for progress in the use of this type of therapy.

In summary, our results suggest that the behavior of cell lines developed under low oxygen conditions (5% O_2_) is generally similar to that of commonly used breast cancer cell lines developed under 21% oxygen conditions. Although no difference in sensitivity to estrogen or doxorubicin was observed between cell lines cultured in 5% vs. 21% oxygen, reduced ROS and upregulation of the hypoxia response, as indicated by *CA9* expression, were observed in cells cultured under 5% oxygen conditions. Therefore we cannot exclude the possibility that culture of cells under 5 vs. 21% oxygen conditions could have consequences for the effectiveness of drug treatment in cell lines as predictors of therapy responses in patients.

## Ethics statement

Ethics approval and consent to participate: This study was carried out in accordance with the recommendations of New Zealand Health and Disability Ethics Committee guidelines–ref AKL/2000/184/AM06, New Zealand Health and Disability Ethics Committee. The protocol was approved by the Northern A Health and Disability Ethics Committee. All subjects gave written informed consent in accordance with the Declaration of Helsinki.

## Author contributions

EYL and BCB designed this study. EYL, CF-P, WRJ, EM, PMK, DCS and MEA-A carried out the study. EYL, CF-P, SKB, RJB, DCS and MEA-A assisted with the data analysis. EYL drafted the manuscript. RJB, GJF, DCS, CF-P, MEA-A, SKB and BCB assisted with the manuscript preparation. All authors read and approved the final manuscript.

### Conflict of interest statement

The authors declare that the research was conducted in the absence of any commercial or financial relationships that could be construed as a potential conflict of interest.

## Supplementary material

Figure S1 Sensitivity of breast cancer cell lines exposed to doxorubicin at 5 or 21% oxygen conditions. IC50 values for doxorubicin shown as the mean ± standard error of triplicate experiments.

Table S1 Short Tandem Repeat profiling for the NZBR cell lines.

Table S2 List of primers used in the experiments.

Table S3 Mutation frequency in breast carcinoma and breast cancer cell lines.

## Abbreviations

ER: estrogen receptor
PR: progesterone receptor
HER2: human epidermal growth factor receptor 2
EGFR: epidermal growth factor receptor
PBS: phosphate-buffered saline
SDS: sodium dodecyl sulfate
TCGA: The Cancer Genome Atlas
GREB1: Growth Regulation By Estrogen In Breast Cancer 1
CCND1: Cyclin D1
TTF1: Trefoil Factor 1
HIF: Hypoxia-inducible factor.
