# Supplementary figures and images for: Derivation of Breast Cancer Cell Lines Under Physiological (5%) Oxygen Concentrations
Source: Front Oncol. 2018 Oct 12;8:425. doi: 10.3389/fonc.2018.00425 (PMC6194255; doi:10.3389/fonc.2018.00425)

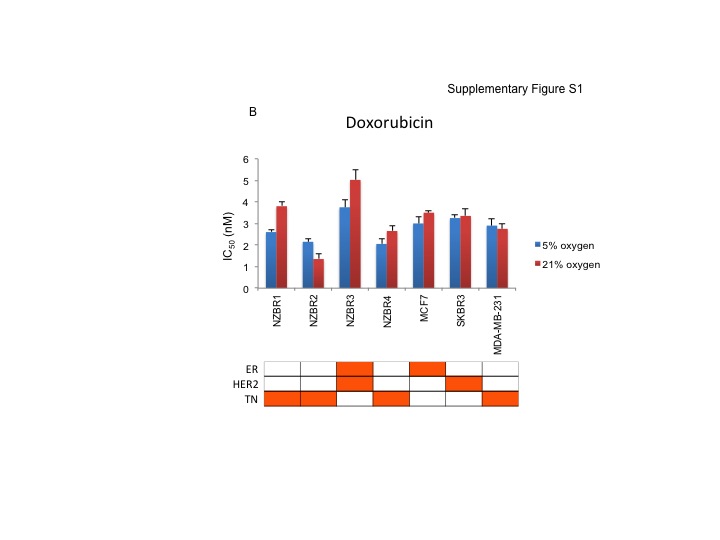

Supplement: Figure S1 — Sensitivity of breast cancer cell lines exposed to doxorubicin at 5 or 21% oxygen conditions. IC50 values for doxorubicin shown as the mean ± standard error of triplicate experiments. [file Image_1.JPEG]
