# Supplementary material for: Derivation of Breast Cancer Cell Lines Under Physiological (5%) Oxygen Concentrations
Source: Front Oncol. 2018 Oct 12;8:425. doi: 10.3389/fonc.2018.00425 (PMC6194255; doi:10.3389/fonc.2018.00425)
Supplement: Table S1 — Short Tandem Repeat profiling for the NZBR cell lines. [file Table_1.DOCX]

Supplementary Table S1. Short Tandem Repeat profiling for the NZBR cell lines

|  |  |  |  |  |
| --- | --- | --- | --- | --- |
|  | **NZBR1** | **NZBR2** | **NZBR3** | **NZBR4** |
| **D8S1179** | 13 | 12,15 | 10,12 | 12,14 |
| **D21S11** | 30 | 29,30 | 32.2 | 30 |
| **D7S820** | 8,10 | 8 | 10,13 | 8,9 |
| **CSF1PO** | 10,12 | 10,12 | 11 | 12 |
| **D3S1358** | 16,19 | 15,17 | 16 | 16 |
| **TH01** | 6,7 | 9 | 9 | 9 |
| **D13S317** | 9 | 8,11 | 11,12 | 14 |
| **D16S539** | 13 | 11 | 11 | 9 |
| **D2S1338** | 19 | 18,21 | 23,24 | 19,24 |
| **D19S433** | 13 | 14,14.2 | 13,14 | 13,14 |
| **vWA** | 14,15 | 14,18 | 18,19 | 14,16 |
| **TPOX** | 8 | 8 | 8,9 | 8,11 |
| **D5S818** | 11 | 7,9 | 11 | 11,13 |
| **D18S51** | 12,15 | 13 | 17 | 11,17 |
| **FGA** | 20,23 | 22,23 | 22,25 | 22 |
| **Amelogenin** | X | X | X | X |
