# Supplementary material for: Derivation of Breast Cancer Cell Lines Under Physiological (5%) Oxygen Concentrations
Source: Front Oncol. 2018 Oct 12;8:425. doi: 10.3389/fonc.2018.00425 (PMC6194255; doi:10.3389/fonc.2018.00425)
Supplement: Table S2 — List of primers used in the experiments. [file Table_2.DOCX]

Supplementary Table S2. List of primers used in the experiments.

| HPRT-F | 5’-TGAGGATTTGGAAAGGGTGT-3’ |
| --- | --- |
| HPRT-R | 5’-GCACACAGAGGGCTACAATG-3’ |
| CA9-F | 5’-CTTGGAAGAAATCGCTGAGG-3’ |
| CA9-R | 5’-TGGAAGTAGCGGCTGAAGTC-3’ |
| GREB1-F | 5’-GGTGGTCTCCAGAATCTCCA-3’ |
| GREB1-R | 5’-CCACCACTGGAAAGGTGACT-3’ |
| CCND1-F | 5’-CCCTCGGTGTCCTACTTCAA-3’ |
| CCND1-R | 5’-AGGAAGCGGTCCAGGTAGTT-3’ |
| TFF1-F | 5’-GTGTGCAAATAAGGGCTGCT-3’ |
| TFF1-R | 5’-GCAGATCCCTGCAGAAGTGT-3’ |
