# Supplementary material for: Derivation of Breast Cancer Cell Lines Under Physiological (5%) Oxygen Concentrations
Source: Front Oncol. 2018 Oct 12;8:425. doi: 10.3389/fonc.2018.00425 (PMC6194255; doi:10.3389/fonc.2018.00425)
Supplement: Table S3 — Mutation frequency in breast carcinoma and breast cancer cell lines. [file Table_3.DOCX]

Supplementary Table S3. Mutation frequency in breast carcinoma and breast cancer cell lines

| **Cell Line** | **Gene** | **Cell Line Frequency** | **Protein Affecting Mutation Frequency** | **Breast Carcinoma Frequency** |
| --- | --- | --- | --- | --- |
|  |  |  |  |  |
|  |  |  |  |  |
| **NZBR2** | **BRCA2** | 14.29% | 1.70% | 4.73% |
|  | **AKT1** | 2.04% | 2.70% | 2.37% |
| **NZBR3** | **NF1** | 14.29% | 2.10% | 2.76% |
|  | **EVI2B** | ND | ND | ND |
|  | **LRP1B** | 22.45% | 1.30% | 0.79% |
| **NZBR4** | **MRE11A** | 4.08% | 0.40% | 0.20% |
|  | **PMS2** | ND | 0.002 | 0.20% |
|  | **MLH1** | 4.08% | 0.20% | 0.20% |
|  | **ATM** | 8.16% | 2.10% | 3.94% |
|  | **BRCA2** | 14.29% | 1.70% | 4.73% |

Frequency of mutated genes from cBioPortal (www.cbioportal.org) using The Cancer Genome Atlas (TCGA) Breast Invasive Carcinoma project (n = 825 cases, Nature 2012) and Cancer Cell Line Encyclopedia (Novartis/Broad, Nature 2012) breast cancer cell lines (n = 56). The protein affecting mutation frequency from IntOGen-mutations platform (www.intogen.org) [60] using breast carcinoma (n = 1148). No mutation of the above gene list was detected in NZBR1. ND, no data.

60. Gonzalez-Perez A, Perez-Llamas C, Deu-Pons J, Tamborero D, Schroeder MP, Jene-Sanz A, et al. (2013) IntOGen-mutations identifies cancer drivers across tumor types. Nat Methods 10: 1081-1082.
